# 27558 Obeticholic acid (OCALIVA ®) protects against 2,8-dihydroxyadenine nephropathy in mice

**DOI:** 10.1017/cts.2021.428

**Published:** 2021-03-30

**Authors:** Bryce Jones, Carlos Benitez, Idalia Cruz, Komuraiah Myakala, Xiaoxin Wang, Andrew Libby, Emma Rowland, Ryan Kurtz, Avi Rosenberg, Moshe Levi

**Affiliations:** 1 Georgetown University; 2 Johns Hopkins University

## Abstract

ABSTRACT IMPACT: This work may lead to new treatments for crystalline nephropathies. OBJECTIVES/GOALS: This study investigated obeticholic acid (OCALIVA ®) as a potential treatment for 2,8-dihydroxyadenine (2,8-DHA) nephropathy using a mouse model. The treatment was investigated in both sexes at two timepoints. METHODS/STUDY POPULATION: Male and female C57BL/6J mice (12 weeks of age) were fed chow (Research Diets D19120401i) or chow admixed with adenine (0.2% w/w) ad lib for either 3.5 or 7 weeks. Mice were treated with either vehicle (corn oil) or obeticholic acid (10 mg/kg BW) by gavage 5 days per week. Each of the 16 combinations of sex/diet/timepoint/treatment groups had an n = 6 (96 mice in total). Food and body weights were measured twice per week, and 24-hour urines were collected prior to euthanasia. Serum and organs were collected and processed for biochemical and histopathological analyses. RESULTS/ANTICIPATED RESULTS: At both the 3.5-week and 7-week timepoints, dietary adenine robustly increased BUN and serum creatinine compared to control diet in vehicle-treated male and female mice (P < .01, all comparisons). At the 3.5-week timepoint, obeticholic acid reduced BUN in male (P < .05) but not female adenine mice. Obeticholic acid did not affect serum creatinine at this timepoint. At the 7-week timepoint, obeticholic acid reduced BUN in female (P < .05) but not male adenine mice. At the 7-week timepoint, obeticholic acid reduced serum creatinine in both male (P < .05) and female (P < .01) mice. Biochemical and histopathological analyses are ongoing, and we anticipate that the results will agree with the serum chemistries. DISCUSSION/SIGNIFICANCE OF FINDINGS: Obeticholic acid is FDA-approved for primary biliary cholangitis, and it is in clinical trials for several other hepatobiliary diseases. Although currently untested in humans, it is nephroprotective in many preclinical models of kidney disease. This study is the first to investigate obeticholic acid in a model of crystalline nephropathy.

